# Immune markers and microbial factors are related with periodontitis severity in people with HIV

**DOI:** 10.1007/s00784-022-04758-6

**Published:** 2022-11-01

**Authors:** Hester Groenewegen, Konstantina Delli, Arjan Vissink, Frederik K. L. Spijkervet, Wouter F. W. Bierman

**Affiliations:** 1 Department of Oral and Maxillofacial Surgery, University of Groningen and University Medical Center Groningen, P.O. Box 30.001, 9700 RB Groningen, the Netherlands; 2 Department of Internal Medicine, Division of Infectious Diseases, University of Groningen and University Medical Center Groningen, P.O. Box 30.001, 9700 RB Groningen, the Netherlands

**Keywords:** HIV, Periodontitis, Inflammation, Immune senescence, Age-related diseases

## Abstract

**Objective:**

The objective of the study is to assess systemic immune markers and microbial factors related to periodontitis severity in people living with HIV.

**Methods:**

Eighty people living with HIV (PLWH), who exhibited in the last two viral load measurements < 40 copies/mL, underwent full-mouth periodontal examinations and sub-gingival plaque sampling. Periodontitis was classified according to the CDC-AAP case definition. Inflammation, immune-activation, and immunosenescence markers were assessed, microbiological analyses were performed, and oral care routines and HIV characteristics were noted.

**Results:**

From our group of PLWH, 42.5% and 57.5% suffered from moderate and severe periodontitis, respectively. Oral care habits did not differ between PLWH with moderate and severe periodontitis. Bacterial subgingival plaque loads were higher, and *Porphyromonas gingivalis* was more prevalent in PLWH with severe periodontitis than with moderate periodontitis (53% vs 7%, respectively). Mean C-reactive protein levels [CRP, 1.6 mg/L versus 0.8 mg/L, *p* = 0.020] and percentages of senescent CD28-CD57 + CD8 + T-cells in peripheral blood [16.5 versus 8.9, *p* = 0.035] were higher with severe periodontitis. Infection duration, CD4 count, CD4/CD8 ratio and type of antiretroviral therapy did not differ between both groups.

**Conclusions:**

Periodontitis severity is related to increased prevalence of *Porphyromonas gingivalis*, elevated CRP levels, and higher frequencies of circulating CD8 + senescent cells in PLWH.

**Supplementary information:**

The online version contains supplementary material available at 10.1007/s00784-022-04758-6.

## Introduction

People with human immunodeficiency virus (HIV) are known to have a higher prevalence of periodontitis compared to the general population [1, 2]. Periodontitis is a chronic disease that is linked to, amongst others, specific oral microorganisms [3]. Periodontitis is also associated with cardiovascular and systemic diseases [4] such as diabetes mellitus and rheumatoid arthritis, as well as with early ageing and age-related diseases [5–7]. In periodontitis, bacteria and bacterial products from the oral biofilm and inflammatory mediators, which are produced locally within the periodontal tissues, enter the blood. This is thought to result in a high inflammatory load reflected by, amongst others, higher levels of CRP [8]. Early periodontitis diagnosis would enable timely therapeutic intervention, reduce tooth loss and, potentially, lower the risk of age-related diseases [9]. Depending on whether a person is undergoing HIV treatment with combination antiretroviral therapy (cART), as well as which periodontal disease classification is used, the prevalence of periodontal diseases in people living with HIV (PLWH) varies between 30 and 100% [2, 10, 11]. PLWH who have periodontitis at the time of HIV diagnosis have a high risk of accelerated periodontal attachment loss over time, compared to seronegative controls [12]. It is not clear whether a HIV infection enhances periodontitis [1, 13].

PLWH are also at a higher risk of developing prematurely age-related diseases, e.g. cardiovascular diseases and diabetes mellitus [14–17]. The driver of this early ageing is believed to be the ongoing systemic immune activation and inflammation. Despite controlled suppression of viral replication under cART, the immune system of PLWH has been shown to age prematurely, leading to immunosenescence [18, 19]. Several studies noted a noticeable relative increase in senescent cell populations in PLWH compared to age-matched controls [20–22], which is linked to systemic immune activation and exhausted CD4^+^ T-cell function [23]. An increase in senescent cells can, in turn, amplify the inflammation [24, 25]. In PLWH, immune activation and systemic inflammation appear to be caused by the translocation of microbial products from the gut mucosal surfaces to the peripheral blood. Microbial translocation could, potentially, lead to systemic inflammation [26]. Current research is focusing almost exclusively on the large intestine as the site of microbial product translocation to the blood [27–29], while other mucosal sites, such as the oral cavity, have rarely been studied. Periodontitis could also be a potential source of microbial translocation to the blood.

A previous study could not find an association between the microbial translocation and immune senescence markers and the size of the periodontal inflammatory surface area (PISA) in PLWH [30]. A possible explanation for this alleged absence of association could be related to the PISA which reflects the surface area of the bleeding pocket epithelium. It is well-known, however, that periodontal pockets might, in themselves, play an important role since they could serve as an area of microbial translocation [31]. Also, it has not been possible to classify patients into groups according to the bleeding and severity of the periodontitis. Consequently, it is challenging to detect patterns and behaviours amongst patients according to their periodontal status. Hence, assessing periodontitis with a different classification system, which is frequently used in epidemiologic studies, that is based more extensively on pocket depth rather than bleeding pocket epithelium, might give more insight.

Thus, the aim of this study was to investigate which markers related to microbial translocation and immune senescence are related to the severity of the periodontitis in PLWH.

## Methods

In a cross-sectional study, PLWH visiting the HIV outpatient clinic of the University Medical Center Groningen (UMCG), the Netherlands, were recruited. Inclusion criteria were age ≥ 18 years, presence of ≥ 6 teeth, use of combination antiretroviral therapy (cART) for ≥ 6 months and the last two viral load measurements yielded < 40 copies/mL. Exclusion criteria were a history of radiation therapy in the head and neck region and an inability to understand spoken or written Dutch or English. The ethics committee of the University Medical Center Groningen (METc number 2014/128) approved this study.

All the participants had to complete a validated health and oral care assessment questionnaire to identify any medical conditions that might be associated with periodontitis, e.g. diabetes and cardiovascular diseases [32–34]. Also, the participants had to complete a questionnaire about their oral health care habits (e.g., brushing frequency, type of toothbrush, interdental cleaning, etc.; additional Table 1). Finally, all the participants were asked if they could indicate with a number how important they rated their dental health on a VAS scale from 0 to 10 (0 indicated not important at all and 10 very important).

**Table 1:** Patient characteristics of the 80 PLWH with moderate and severe periodontitis

		Total population	People with moderate periodontitis (*n* = 34)	People with severe periodontitis (*n* = 46)	*p* value
Male, *N* (%)		69 (86.3)	27 (79.4)	42 (91.3)	0.127
Mean age in years (SD)		50.8 (11.4)	50.6 (11.9)	51.0 (11.1)	0.891
Mean BMI (kg/m^2^) (SD)		24.3 (3.0)	23.9 (3.1)	24.5 (2.8)	0.317
Tobacco use, *N* (%)	Current smoker	22 (27.5)	6 (17.6)	16 (34.8)	0.227
	Never smoked	32 (40)	16 (47.1)	16 (34.8)	
	Former smokers	26 (32.5)	12 (35.3)	14(30.4)	
People with diabetes mellitus, *N* (%)		6 (7.5)	2 (5.9)	4 (8.7)	0.637
History of cardiovascular disease, *N* (%)		17 (23.6)	8 (25.8)	9 (22.0)	0.703

### Periodontal assessment

The eligible participants underwent full-mouth periodontal examinations by an experienced dental hygienist (H.G.). The periodontium of all the teeth was examined with a periodontal probe (Williams probe 14 W, Hu-Friedy Mfg. Co., LLC, UK). Bleeding on probing (BoP), probing pocket depth (PPD) and clinical attachment level (CAL) were measured at six sites per tooth. The number of missing teeth was recorded. The presence of periodontitis was defined according to the CDC-AAP case definition of periodontitis surveillance for epidemiologic studies. Mild periodontitis was recorded for cases with ≥ 2 interproximal sites with a CAL ≥ 3 mm and ≥ 2 interproximal sites with a PPD ≥ 4 mm (not on the same tooth) or 1 site with a PPD ≥ 5 mm. The participants were classified as having moderate periodontitis in the presence of ≥ 2 interproximal sites with a CAL ≥ 4 mm (not on the same tooth) or ≥ 2 interproximal sites with a PPD ≥ 5 mm, also not on the same tooth. Severe periodontitis was recorded if the participants had ≥ 2 interproximal sites with clinical attachment loss, a CAL ≥ 6 mm, not on the same tooth, and ≥ 1 interproximal site with a PPD ≥ 5 mm [35–37].

Periodontal (sub-gingival) plaque samples were taken from each quadrant. The paper points were inserted to the depth of the pockets from the deepest bleeding pocket in each quadrant of the dentition and consequently left in place for 10 s and pooled in 2 ml of reduced transport fluid. Microbiological analysis of the samples was performed by the Oral Microbiology Laboratory of the UMCG, according to standard culturing protocol [38, 39]. The outcome variables of the microbiological analyses were the presence of the periodontal pathogens *Aggregatibacter actinomycetemcomitans (Aa)*, *Porphyromonas gingivalis (Pg)*, *Prevotella intermedia (Pi)*, *Tannerella forsythia (Tf)*, *Parvimonas micra (Pm)*, *Fusobacterium nucleatum (Fn)*, *Campylobacter rectus (Cr)*, and the total anaerobic viable count (TVC) [40]. Participants who had used antibiotics in the last 3 months were excluded from the microbiological analysis.

### Flow cytometric analyses of immune parameters

Venous blood was drawn to measure the absolute numbers of CD3^+^, CD4^+^ and CD8^+^ T-cells and measured using the MultiTest TruCount method with MultiTest reagents directed at CD45/3/4/8 (Becton Dickinson). To assess if periodontitis accelerates immune senescence, a variety of immune parameters identifying naïve (CD45R0^−^CCR7^+^ CD28^+^), senescent (CD28^−^CD57^+^) and activated (HLA-DR^+^ or CD38^+^) subsets within the CD4^+^ and CD8^+^ T-cells were employed. The viral load measurement was performed on EDTA plasma samples using the Abbott Real-Time HIV-1 assay. The participants were classified according to the ‘Revised Surveillance Case Definition for HIV-Infection’ in three stages, based on the CD4 T-lymphocyte count or the presence of opportunistic illness [41]. Additional information was collected about the mode of HIV-transmission, years of HIV-infection, type of cART and current CD4^+^/CD8^+^, CD4^+^ nadir levels.

### ELISA assessment of soluble markers

Serum concentrations of the inflammation markers CRP, IL-6 and CXCL-10 and the microbial translocation and inflammation markers sCD14, LPS and sCD163 were assessed with ELISA. IL-6, LPS, CXCL-10 (R&D systems, Minneapolis, MN), sCD-14 and sCD-163 (Thermo Scientific, Waltham, MA) were measured according to the Dynex DS-2 system manufacturer’s protocol. High-sensitivity CRP levels were measured and determined using Cobas (Roche Diagnostics).

Missing data were completed from the patient chart and by searching the database of the national HIV-monitoring Foundation (SHM), which is the executive organization for the registration and monitoring of consenting participants with HIV-infection for care in the 27 Dutch HIV-treatment centres [42].

### Statistical analysis

Q–Q plots were used to determine the distribution of the data. The qualitative and quantitative features of PLWH with severe periodontitis and PLWH with moderate periodontitis were compared by means of a Fisher exact/Chi-square test and independent samples *t* tests or a Mann–Whitney *U* test, when appropriate. All the analyses were performed using the SPSS software, version 23 (SPSS, Chicago, IL, USA).

## Results

### Patients

**Fig. 1:**
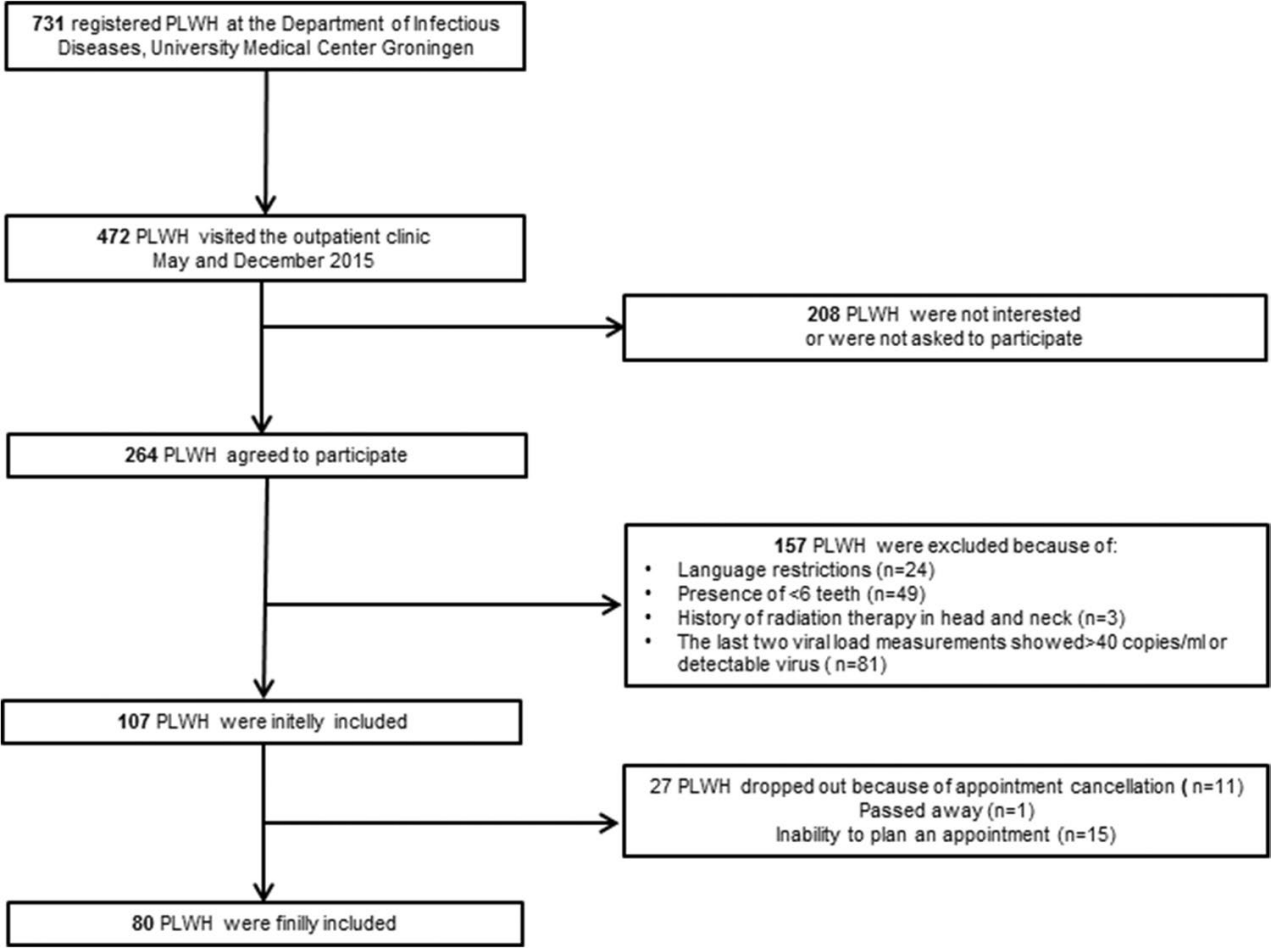
Flow chart of patient inclusion

All the included participants’ characteristics are shown in Table 1. From the initial 472 who visited the outpatient clinic, 264 participants accepted the invitation to participate in this study. Of these, 184 participants had to be excluded for several reasons (Fig. 1), resulting in 80 participants being eligible for the study. All the included participants were diagnosed with moderate (42.5%) or severe (57.5%) periodontitis; no one was diagnosed with no periodontitis or mild periodontitis. There were no significant differences between the severe and moderate periodontitis groups regarding gender, age, diabetes, and cardiovascular diseases (Table 1).

### Periodontal measurements

The oral characteristics of the participants are described in Table 2. BoP was significantly higher in participants with severe periodontitis (46.7%), compared to moderate periodontitis (34.7%, *p* = 0.001). None of the reported oral care habits were significantly different between both groups. *Pm* was detected in all the participants with severe periodontitis and in 89.7% of the group with moderate periodontitis (*p* = 0.042). *Pg* was much more prevalent in the participants with severe periodontitis (21 participants, 45.7%) than in participants with moderate periodontitis (two participants, 5.9%, *p* < 0.001). *Aa* was only present in 4 participants (8.7%) with severe periodontitis and in none of the participants with moderate periodontitis (*p* = 0.780). The presence of *Fn* was not significantly different between the two groups. *Pm* was present in 95% and *Fn* in 94% of all the participants, making them the most frequently detected bacteria in both groups.

**Table 2:** Oral care factors of the 80 PLWH with moderate and severe periodontitis

		Total population (*n* = 80)	People with moderate periodontitis (*n* = 34)	People with severe periodontitis (*n* = 46)	*p* value
Mean probing pocket depth, mm (PPD, SD)		3.1 (0.5)	2.8 (0.3)	3.4 (0.5)	0.000
Mean cemento-enamel junction, mm (CEJ, SD)		1.5 (0.3)	1.4 (0.2)	1.6 (0.4)	0.006
Mean clinical attachment level, mm (CAL, SD)		4.6 ( 0.7)	4.2 (0.3)	5.0 (0.7)	0.000
Bleeding on probing, % (BoP, SD)		41.6 (17.0)	34.7 ( 14.7)	46.7 (16.9)	0.001
Dentist awareness of HIV infection*, %, *N* (75)	No	25 (33.3)	11 (34.4)	14 (32.6)	0.878
Dental wishes of the patient: Patient wishes to maintain teeth**	Yes	75 (96.2)	32 (97.0)	43 (95.6)	0.748
Periodontal/gum diseases treated	Yes	38 (47.5)	13 (34.2)	25 (65.8)	0.154
Frequency of brushing teeth (per day)	Not daily	3 (3.8)	1 (2.9)	2 (4.3)	0.339
	Daily	14 (17.5)	3 (8.8)	11 (23.9)	
	Twice a day	54 (67.5)	26 (76.5)	28 (60.9)	
	More than twice a day	9 (11.3)	4 (11.8)	5 (10.9)	
Use of interdental cleaning materials	Yes	70 (87.5)	29 (85.3)	41 (89.1)	0.608
Did not use antibiotics in the last 3 months		67	29(43.3)	38 (56.7)	0.748
Presence of *Aggregatibacter actinomycetemcomitans*,* N* (%)		4 (5)	0 (0.0)	4(8.7)	0.078
Presence of *Porphyromonas gingivalis*, *N* (%)		23 (28.8)	2 ( 5.9)	21 (45.7)	< 0.001
Presence of *Prevotella intermedia*, *N* (%)		61 (76.3)	27 (79.4)	34(73.9)	0.568
Presence of *Tannerella forsythia*, *N* (%)		44 (55.0)	18 (52.9)	26 (56.5)	0.750
Presence of *Parvimonas micra*, *N* (%)		76 (95.0)	31 (91.2)	45 (97.8)	0.042
Presence of *Fusobacterium nucleatum*, *N* (%)		76 (95.0)	32 (94.1)	44 (95.7)	0.756
Presence of *Campylobacter rectus*, *N* (%)		25 (31.3)	11 (31.0)	14 (30.4)	0.885
Mean bacterial load (SD)		1.81E + 008 (2.41E + 008)	9.32 E + 007 (1.77 E + 008)	2.25 E + 008 (2.51E + 008)	0.006
Median VAS regarding importance of dental health (IQR)		9.0 (8–10)	9 (8–10)	9 (8–9)	0.065

^*^This question was applicable to only 75 people who stated they had visited a dentist

^**^Data available for 78 people

### Bacterial translocation, inflammation and immunosenescence

Infection duration, CD4 count and CD4/ CD8 ratio, as well as type of cART, were not significantly different between the severe periodontitis and moderate periodontitis groups (Table 3). We did not detect higher LPS or sCD14 levels as markers of bacterial translocation in PLWH with severe periodontitis, nor did we detect an elevation of systemic IL-6, sCD163, CXCL10 as markers of inflammation in PLWH with severe periodontitis. Only the CRP levels were significantly higher in the group with severe periodontitis than in the group with moderate periodontitis [1.6 mg/L (IQR 0.9–3.0) versus 0.8 mg/L (IQR 0.5–2.0), respectively, *p* = 0.02] (Table 4).

**Table 3:** HIV related characteristics of the 80 PLWH with moderate and severe periodontitis

		N (%) Mean (SD) or median (IQR)	People with moderate periodontitis (*n* = 34)	People with Severe periodontitis (*n* = 46)	*p* value
Type of cART	PI-based	19 (23.8)	10 (29.4)	9 (19.6)	0.379
	NNRT-based	42 (52.5)	18 (52.9)	24 (52.2)	
	INT-based	12 (15.0)	5 (14.7)	7 (15.2)	
	Others	7 (8.8)	1 (2.9)	6 (13.0)	
CDC classification	Stage 1	9 (11.3)	4 (11.8)	5 (10.9)	0.987
	Stage 2	36 (45.0)	15 (44.1)	21 (45.7)	
	Stage 3	35 (43.8)	15 (44.1)	20 (43.5)	
CD4^+^ nadir *	< 200 T-cells/mm^3^	28 (35.0)	11 (33.34)	17 (39.5)	0.692
	200- < 500 T-cells/mm^3^	41 (51.3)	18 (52.9)	23(53.5)	
	> 500 T-cells/mm^3^	7 (8.8)	4 (12.1)	3 (7.0)	
CMV positive		74 (92.5%)	31 (91.2%)	43 (93.5%)	0.200
Duration of infection (years)		9.4 (6–15)	9.3 (6–14)	9.5 (7–16)	0.480^#^
Duration of cART use		9.5 (6.1)	9.3 (5.8)	9.7 (6.4)	0.793
CD4^+^/CD8^+^(T-cells/mm^3^) *		0.9 (0.6–1.3)	0.97 (0.8–1.3)	0.86 (0.5–1.3)	0.186^#^
CD4^+^ nadir (T-cells/mm^3^) *		225.0 (123–340)	218.0 (145–350)	230.0 (120–320)	0.593^#^

^*^CD4^+^ nadir was not known in 4 people due to incomplete patient charge information^+^

^#^ Mann–Whitney *U* test applied due to skewed data distribution

**Table 4:** HIV and immunologic related characteristics of the 80 PLWH with moderate and severe periodontitis

	Mean (SD) or median (IQR)	Participants with moderate periodontitis (*n* = 34)	Participants with severe periodontitis (*n* = 46)	*P* value
CD4 + (cells/mm^3^)	670.3 (273.7)	706.7 (274)	643.3 (273.1)	0.309
Naive CD4 + T cells (% of CD4 + T cells)	26.8 (14.3)	28.6 (15.1)	25.5 (13.7)	0.344
CD4 + HLA-DR + T cells (% of CD4 + T cells)*	14.3 (11–26)	13.0 (10–21)	15.0 (12–30)	0.124^#^
CD4 + CD38 + T cells (% of CD4 + T cells)	45.1 (15.2)	47.3 (15.0)	43.5 (15.2)	0.266
CD4 + HLA-DR + + CD38 + T cells (% of CD4 + T cells)*	4.6 (4–8)	4.7 (3–8)	4.5 (4–8)	0.685^#^
CD4 + CD28-CD57 + T cells (% of CD4 + T cells)	6.6 (6.1)	5.9 (4.2)	7.1 (7.2)	0.331
CD3 + (cells/mm^3^)	1428.5 (1165–1785)	1403.0 (1154–1732)	1461.0 (1147–1796)	0.640^#^
CD8 + (cells/mm^3^)	695.5 (523–884)	681.0 (456–808)	701.0 (559–957)	0.411^#^
Naive CD8 + T cells (% of CD8 + T cells)	10.3 (5–19)	9.8 (5–18)	10.1 (5–20)	0.992^#^
CD8 + HLA-DR + T cells (% of CD8 + T cells)*	22.9 (18–36)	22.8 (17–33)	22.9 (18–40)	0.751^#^
CD8 + CD38 + T cells (% of CD8 + T cells)	29.6 (19–38)	28.3 (20–40)	30 (18–35)	0.400^#^
CD8 + HLA-DR + + CD38 + T cells (% of CD8 + T cells)*	8.1 (5–13)	8.6 (6–13)	7.7 (5–12)	0.392
CD8 + CD28-CD57 + T cells (% of CD8 + T cells)	11.4 (7–23)	8.9 (6–19)	16.5 (9–26)	0.035^#^
CRP (mg /l)	1.2 (0.6–2.3)	0.8 (0.5–2.0)	1.6 (0.9–3.0)	0.020^#^
IL-6 (pg/ml)	0.7 (0.0–1.8)	0.6 (0.0–1.5)	0.9 (0.0–2.6)	0.211^#^
sCD14 (µg/ml)	3.9 (1.3)	3.6 (1.3)	4.1 (1.3)	0.079
sPD-1 (pg/ml)	204.8 (163–183)	208.4 (176–260)	184 (162–250)	0.243^#^
CXCL10* (pg/ml)	147 (118–183)	136.4 (116–173)	155.1 (118–190)	0.507^#^
sCD163 (ng/ml)	34.0 (28–50)	39.5 (26–57)	33.8 (29–45)	0.853^#^
LPS/DPLG70 (ng/ml)	136 (46.3)	139.0 (50.3)	133.7 (43.5)	0.623

^*^data was not known for 1 person

^#^Mann–Whitney *U* test applied due to skewed data distribution

In the participants with severe periodontitis, the median percentage of senescent CD28^−^CD57^+^CD8^+^ T-cells was significantly higher than in those with moderate periodontitis (16.5 versus 8.9%, respectively, *p* = 0.03). In addition, we found a significant positive correlation between the levels of CD28^−^CD57^+^CD8^+^ T-cells and levels of sCD14 (*r* = 0.3, *p* = 0.012), Together, these results suggest that higher levels of senescent CD28^−^CD57^+^CD8^+^ T-cells may be attributed to microbial translocation in severe periodontitis. We found no correlation between the levels of CD28^−^CD57 ^+^ CD8^+^ T-cells and LPS. Inflammation markers (e.g., CRP and IL-6) were not related to higher levels of CD28^−^CD57^+^CD8^+^ T-cells.

## Discussion

We aimed to assess whether systemic immune markers and microbial factors are related to periodontitis severity in PLWH. In this study, we found increased levels of CRP, increased frequencies of CD28^−^CD57^+^CD8^+^ T-cells and a higher prevalence of *Pg* and *Pm* in PLWH with severe periodontitis.

The more elevated CRP levels in the severe periodontitis participants compared to the moderate periodontitis participants are in line with the results of the Paraskevas et al. meta-analysis [8]. This meta-analysis showed that in a population of non-HIV participants with periodontitis, a high inflammatory load was reflected by higher levels of CRP. A small increase in CRP levels is commonly regarded as an indication of low-grade inflammation, which periodontitis, per definition, is [8]. Elevated CRP levels are considered to be a risk factor for developing cardiovascular diseases in the general population. As a matter of fact, the relative risk of developing cardiovascular diseases falls into three major categories, i.e. low, average and high, based on the systemic CRP levels. Specifically, CRP levels of < 1.0, 1.0 to 3.0 and > 3.0 mg/L correspond to low, average and high risk, respectively [43]. In our study, the PLWH with severe periodontitis had a median CRP of 1.6 mg/L and so, according to the aforementioned categorization, would have an average relative risk of cardiovascular diseases, while the PLWH with moderate periodontitis, with a CRP of 0.8 mg/L, would have a low relative risk. The higher CRP levels in the group with severe periodontitis could be attributed to more periodontal inflammation. Periodontal treatment by dental professionals predictably reduces periodontal inflammation and probing pocket depths and thus periodontitis severity [44]. Consequently, professional periodontal therapy could decrease the CRP levels and potentially lower the risk of cardiovascular disease [45].

We found higher percentages of senescent CD28^−^CD57^+^CD8^+^ T-cells in the severe periodontitis group. While it is known that older adults are characterized by a proportional accumulation of senescent CD28^−^CD57^+^CD8^+^ T-cells compared to younger adults, in our study, the mean age did not differ between the PLWH with severe and moderate periodontitis; thus, age is presumably not a contributing factor here. An increase in CD28^−^CD57^+^CD8^+^ T-cells has also been observed in individuals with other chronic viral infections [46]. CD28^−^CD57^+^CD8^+ ^T-cells are believed to approach end-stage senescence [46]. Moreover, in general, nearly all HIV-infected people are also infected with cytomegalovirus (CMV) [47, 48]. Several studies demonstrated that a persistent CMV infection, possibly acquired prior to HIV, also plays a substantial role in accelerating immunosenescence [47, 49]. Since almost all the participants in our cohort (92.5%) were infected with CMV, CMV infection alone is not likely to have been responsible for the difference in the proportions of senescent CD8^+^ T-cells between the study groups. Next to CMV, inflammation is well known to accelerate immunosenescence and to contribute to higher percentages of CD28^−^CD57^+^ CD8^+ ^T-cells [46, 50]. We did not find a significant relationship between senescent CD4^+^ T-cells and periodontitis severity. Another study showed that senescent CD4^+^ T-cells decline after starting a successful cART, while the percentage of senescent CD8^+^ T-cells does not decrease [51]. The fact that all our study subjects had been on cART for a mean period of 9.5 years might explain the link found between CD8^+ ^T-cells and periodontitis severity.

Another finding is the positive relationship between the presence of the oral bacteria *Pm* and *Pg* and severe periodontitis. Although *Pm* is part of the normal commensal flora, it is significantly more present in patients with severe/moderate periodontitis than in patients with good periodontal health, gingivitis and/or mild periodontitis [52, 53]. Similarly, in the general population, the prevalence of *Pg* is significantly higher in subjects with severe periodontitis than in subjects with good gingival health or various degrees of gingivitis [54, 55]*. Pg* can produce a large amount of butyric acid as a metabolite, which might collapse the homeostasis in the periodontal tissues, and so contribute to the progression of periodontitis [56]. *Pg* could also induce HIV-1 reactivation since butyric acid has been found to be responsible for reactivating latent HIV virus [57, 58]. The levels of butyric acid were, however, not investigated in our study’s participants. Consequently, any reactivation in those individuals due to the presence of butyric acid, or due to the translocation of microbial products from the blood, should be interpreted with caution.

Our study has some limitations. The study is explorative in nature, so we cannot exclude that the relationship between severe periodontitis with percentages of senescent CD28^−^CD57^+^CD8^+ ^T-cells and the prevalence of *Pg* is a chance finding. Due to the explorative character of the study, a calculation of the minimum sample size was not performed, and this may have, in part, influenced the findings. This is why our results should be seen as hypothesis generating, not as definitive findings. Another limitation of our study is that only subjects with severe and moderate periodontitis were compared. Therefore, in future studies, the results should be confirmed in subjects with mild periodontitis and in subjects with non-HIV periodontal diseases. Next, as with any periodontal screening instrument, the one used herein has its limitations too. The CDC-AAP case definition classification used by epidemiologic studies to survey periodontitis [35–37] is based on PPD and CAL. PLWH are known to have more permanent gingival or periodontal tissue loss compared to non-HIV periodontal patients because of necrotizing ulcerative gingivitis or necrotizing ulcerative periodontitis in the past [13]. These conditions are usually seen in patients with a compromised immune system. The severity of periodontitis in patients with irreversible attachment loss, possibly obtained before starting cART, might be overestimated by this classification system. Nevertheless, the CDC-AAP case definition classification is the best method as the measurements are extensive, and, as it is commonly used, the results can be compared. An early diagnosis of periodontitis in PLWH might allow for a timely therapeutic intervention by oral health professionals. Consequently, less periodontal inflammation could have a positive effect on the oral health and thereby could probably reduce the risk of age-related diseases.

## Conclusion

In our study, we observed that severe periodontitis in PLWH is related to higher levels of CRP, higher levels of *Pg* and higher proportions of senescent CD8 + T-cells. This might suggest that severe periodontitis in PLWH could contribute to a higher risk of developing age-related inflammatory conditions such as cardiovascular and systemic diseases.

## Supplementary information

Below is the link to the electronic supplementary material.

Supplementary file1 (DOCX 23 KB)
