# Supplementary material for: Immune markers and microbial factors are related with periodontitis severity in people with HIV
Source: Clin Oral Investig. 2022 Nov 1;27(3):1255–63. doi: 10.1007/s00784-022-04758-6 (PMC9985580; doi:10.1007/s00784-022-04758-6)
Supplement: Supplementary file 1 — Supplementary file1 (DOCX 23 KB) [file 784_2022_4758_MOESM1_ESM.docx]

**Supplementary Table 1: English version of the questionnaire used to assess the health status and the oral health awareness of people with HIV-1 infection.**

**Date of Visit:**

**1. Are you taking any medication?** yes □ (go to question1a) / no □ (go to question 2)

1a. What are you taking and why?

*Medication Indication*

......................................... ………………………………………………………

……………………………. ………………………………………………………

……………………………. ………………………………………………………

……………………………. ………………………………………………………

……………………………. ………………………………………………………

……………………………. ………………………………………………………

**2. Do you have diabetes?** yes □ (go to question 2a/b) / no □ (go to question 3)

2a. Do you use insulin? yes □ / no □

2b. Are you well regulated? yes □ / no□ / unknown □

**3.** **Have you been associated with cardiovascular diseases / thrombosis?**
 yes □ / no□ / unknown □

**4. Do you smoke?** yes □ (go to question4a/b)

no □ smoked in the past □ (go to question 5)

no □ never smoked □ (go to question 5)

4a. What do you smoke?

□ Cigarettes

□ Shag

□ Pipe

□ Other, namely. ……………………………………………………………………………...

4b. How much do you smoke a day? ………………………

**5. Do you go to a dentist?**

o yes. I go regularly (go to question 5a)

o yes. But only in case of complaints (go to question 5b)

o no (go to question 5b)

5a. How often do you go to the dentist? ............ times a year

5b. How long has it been since you last visited a dentist?

……………… months / years

5c. Is your dentist aware of your HIV? yes □ / no □

**6.** **Are you also going to a dental hygienist or are your gums being treated elsewhere**

**(periodontitis)?**

yes □ (go to question 6a/b) / no □ (go to question 7)

Do you go to a: *(multiple answers possible)*

□ Dental hygienist

□ Prevention Assistant

□ Periodontologist

□ Other, namely: .............................................

6b. How often do you go there?

To the .............................................. Patient goes ............... ... times a year
To the .............................................. Patient goes ............... ... times a year

To the .............................................. Patient goes ............... ... times a year

**7 Can you indicate with a number how important your teeth are on a scale of 0 to 10? 0 is not important at all and 10 very important.**

**0 1 2 3 4 5 6 7 8 9 10**

**8. What are your wishes regarding your teeth?**

Do you want to keep your own teeth as long as possible? yes □ / no □

Do you wish to get all your teeth extracted and get a denture? yes □ / no □

If it is necessary for all your teeth to be extracted, would you accept that? yes □ / no □

**9. Do you brush your teeth?** Yes □ (go to question 9a/b) / No □ (go to question 10)

9a. How do you brush your teeth?

□ Electric brush only (go to question 9b)

□ Manual brush only (go to question 9b)

□ Both (go to question 9b)

9b. How often do you brush your teeth?

□ not daily

□ daily

□ twice a day

□ more than twice a day

□ other, namely:……………………………………………………………………………

**10.** **Do you also clean the spaces between the teeth?**

yes □ (go to question 10a/b) / no □ (go to question 11)

10a. What do you use to clean the spaces between the teeth (*multiple answers possible*)*?*

□ dental floss

□ toothpicks

□ interdental brushes

□ Other, namely:…………………………………………………………………………….

10 b. How often do you clean the spaces between your teeth?

□ once a week

□ every other day

□ daily

□ more than once a day

11. Do you use a mouthwash?

yes o (go to question 11a/b) / no o (go to question 12)

11a. What kind of mouthwash do you use (multiple answers possible)

o Chloorhexidine

o Elmex

o Listerine

o Meridol

o Salt-soda

o Other, namely.…………………………………………………………………….

11b How often do you use mouthwash? (Per type; write the type of mouthwash there).

o once a week type:……………………………………….

o every other day type:……………………………………….

o daily type:……………………………………….

o more than once a day type:………………………………………..

**12. Do you have any removable dentures?**

yes □ (go to question 12a) / no □

12a. *Mark what is applicable*

**Upper denture Lower denture**

□ partial prostheses □ partial prostheses

□ complete prostheses □ complete prostheses

□ denture on implants □ denture on implants

□ other:…………………… □ other:……………………
